# A diagnostic LAMP assay for rapid identification of an invasive plant pest, fall armyworm *Spodoptera frugiperda* (Lepidoptera: Noctuidae)

**DOI:** 10.1038/s41598-021-04496-x

**Published:** 2022-01-21

**Authors:** Arati Agarwal, Lea Rako, Mark K. Schutze, Melissa L. Starkie, Wee Tek Tay, Brendan C. Rodoni, Mark J. Blacket

**Affiliations:** 1 Agriculture Victoria, AgriBio, 5 Ring Road, Bundoora, VIC 3083 Australia; 2 Biosecurity Queensland, Queensland Department of Agriculture and Fisheries, GPO Box 267, Brisbane, QLD 4001 Australia; 3 CSIRO, Black Mountain Laboratories, Clunies Ross Street, Canberra, ACT 2601 Australia; 4 Applied BioSciences, University of Macquarie, Sydney, NSW 2109 Australia; 5 School of Applied Systems Biology, La Trobe University, Bundoora, VIC 3083 Australia

**Keywords:** Molecular biology, Zoology

## Abstract

Fall armyworm (FAW), *Spodoptera frugiperda* (Lepidoptera: Noctuidae), is a highly polyphagous invasive plant pest that has expanded its global geographic distribution, including recently into much of Australia. Rapid diagnostic tests are required for identification of FAW to assist subsequent management and control. We developed a new loop-mediated isothermal amplification (LAMP) assay based on the mitochondrial cytochrome c oxidase subunit I (COI) gene for accurate and timely diagnosis of FAW in the field. The specificity of the new assay was tested against a broad panel of twenty non-target noctuids, including eight other *Spodoptera* species. Only *S. frugiperda* samples produced amplification within 20 min, with an anneal derivative temperature of 78.3 ± 0.3 °C. A gBlock dsDNA fragment was developed and trialled as a synthetic positive control, with a different anneal derivative of 81 °C. The new FAW LAMP assay was able to detect FAW DNA down to 2.4 pg, similar to an existing laboratory-based real-time PCR assay. We also trialled the new FAW assay with a colorimetric master mix and found it could successfully amplify positive FAW samples in half the time compared to an existing FAW colorimetric LAMP assay. Given the high sensitivity and rapid amplification time, we recommend the use of this newly developed FAW LAMP assay in a portable real-time fluorometer for in-field diagnosis of FAW.

## Introduction

Fall armyworm (FAW), *Spodoptera frugiperda* (Lepidoptera: Noctuidae), is a highly polyphagous invasive plant pest that is rapidly expanding its distribution worldwide. Native to the Americas, it was first reported in West and Central Africa in 2016^1^, and has since been confirmed across Asia^2–4^ and Oceania^5^ and is now present in over 70 countries^6^. In early 2020, FAW was detected in northern Australia and was determined to be ineradicable in that region^7^. Despite this, efforts are still being made to detect outbreaks early to aid management^8^, as this species poses a major threat to food security worldwide^9^.

Fall armyworm has high fecundity, can rapidly develop resistance to insecticides^10–12^, utilises a wide range of host plants, and has the capacity to migrate long distances, characteristics which have allowed it to rapidly disperse and establish in exotic regions^13–17^. Moths have been observed migrating as far as 1600 km in 30 h with the assistance of wind^18^. Moreover, FAW has been reported from more than 350 plant species^19^, comprising over 80 commercial crops including maize, cotton, sorghum, rice and sugarcane, although whether completion of developmental cycle on these diverse host plants by larvae was possible remains poorly understood. If left unmanaged, FAW has the potential to destroy crops overnight^20,21^.

Morphologically, FAW is very similar to close relatives, especially congenerics; with confident diagnosis of adult (moth) specimens typically reliant on dissection and microscopic examination of male genitalic structures^22,23^. Female moths, therefore, are often impossible to identify, especially if in poor condition with loss of wing scales. Larvae (caterpillars) are similarly problematic, bearing close resemblance to other noctuids, with early-instars being particularly difficult to identify^22,23^.

Because FAW can cause such devastation in a short period of time, there is a global need for a rapid molecular diagnostic test to assist with early and accurate incursion responses. Some of the current methods used to identify FAW include restriction fragment length polymorphism (RFLP)^24^; PCR and Sanger sequencing of both COI^25^ and TPI^20,26^ gene regions; species-specific multiplex PCR primers^27^; and real-time PCR assays^28^. These techniques, while effective at diagnosing the target insect, are time-consuming and are often expensive, requiring highly specialized laboratory facilities and expert staff. A potential solution to these issues, including decreasing the time to reach diagnosis, is loop-mediated isothermal amplification (LAMP)^29^. LAMP assays are quick, simple, low-cost, and have been successfully deployed for diagnosing other plant pests such as the Queensland fruit fly *Bactrocera tryoni*^30^, grape phylloxera *Daktulosphaira vitifoliae*^31^, and Khapra beetle *Trogoderma granarium*^32^ in the field. Given the success of this method across different pest species^30,33–35^, its relative ease of implementation, and the need for rapid diagnosis of FAW following a suspected incursion, LAMP is a highly suitable in-field diagnostic tool to be used in tracking its occurrence in supporting its management. gBlocks Gene Fragments are targeted synthetic oligonucleotide dsDNA which can be used as standards in qPCR reactions^36^, and can be used to provide reliable positive controls for LAMP reactions^31,32^. A gBlock fragment as a standard provides comparable sensitivity, reliability, and assay performance to a purified amplicon standard.

Recently a LAMP assay was published for identification of FAW, targeting a tRNA region of the mitochondrial genome^37^. This assay involves colorimetric detection to identify positive samples through extended incubation of LAMP reactions for 1 ½ hours. This assay can be conducted using relatively simple technology (i.e., a heat block), to induce a colour change from pink to yellow in positive samples. However, it has not been tested using alternative commercially available LAMP master mixes suitable for use on portable real-time fluorometers, which are commonly used for LAMP assays in the field. A second LAMP assay has been very recently developed and optimised for in-field use for larval diagnostics^38^. This assay targets the mitochondrial 5′-COI locus, commonly used for DNA barcoding, in a four-primer LAMP assay system.

This study aimed to develop, and laboratory validate a LAMP assay for accurate and timely diagnosis of FAW for use in the field. The main aims of this study were to: (i) develop an alternative LAMP assay based on mitochondrial cytochrome c oxidase subunit I (3′-COI) gene sequences from multiple *Spodoptera* species to compare with the recently published^37^ assay; (ii) design and evaluate a gBlock dsDNA fragment for use as a reliable FAW DNA positive control in the new LAMP assay; (iii) compare the new LAMP assay with the existing real-time PCR method of FAW identification; (iv) validate the existing colorimetric FAW LAMP assay previously developed by Kim, et al.^37^ on FAW DNA samples through comparison of both the published colorimetric detection method and on a portable real-time fluorometer.

## Results

### Molecular variation

**Figure 1:**
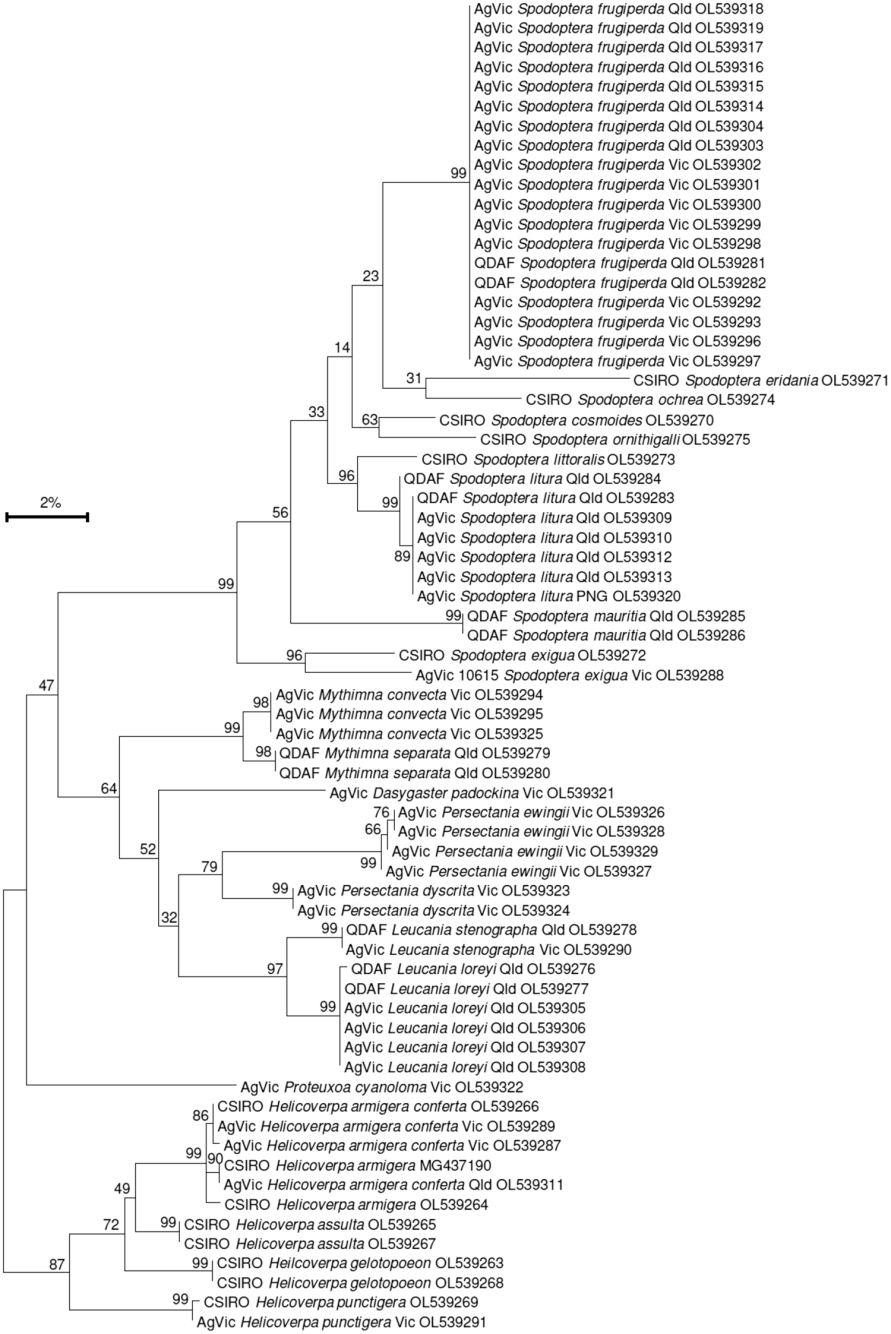
Maximum Likelihood tree (5′-COI DNA sequences) of samples used for testing FAW LAMP assay. Bootstrap values indicated on nodes. AgVic, Agricultural Victoria; CSIRO, Commonwealth Scientific and Industrial Research Organisation; QDAF, Department of Agriculture and Fisheries Queensland; Vic, Victoria Australia; Qld, Queensland Australia; PNG, Papua New Guinea.

The panel of species tested included eight non-target *Spodoptera* species which were between 4.3%, and 7.4% divergent (5′-COI, uncorrected *p*-distances) from FAW, while the other twelve Noctuidae species tested were from 9.3 to 11.2% divergent from FAW (Fig. 1). All new target and non-target new DNA barcode sequences obtained in the current study have been submitted to GenBank (OL539263 - OL539329).

### FAW LAMP assay design and optimisation

**Figure 2:**
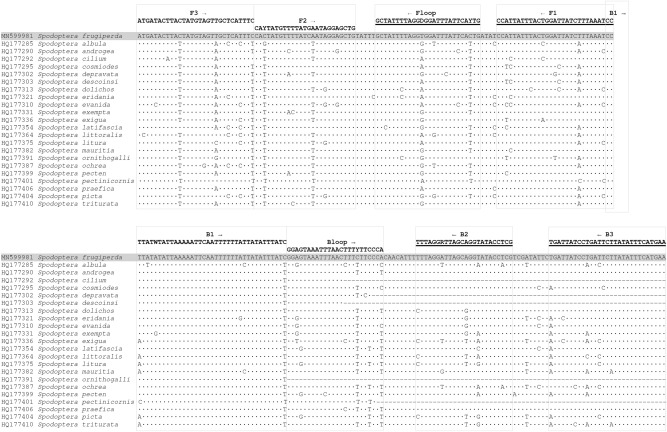
Mitochondrial COI DNA sequence (3′ region) alignment showing FAW LAMP primers. Sequence of FAW (grey shading, from Kim et al*.*^37^) and other closely related *Spodoptera* species^20,55^ obtained from GenBank. Reverse primers are underlined; FIP (5′-3′) is made by combining F1 (reverse compliment) and F2; BIP (5′-3′) is made by combining B1 and B2 (reverse compliment).

LAMP primers (Table 1) were developed to target a 249 bp portion of the FAW COI locus (3′ region) which has been shown to be highly variable in numerous *Spodoptera* species (Fig. 2). Ambiguous bases were added to primers (Fig. 2, Table 1) to account for genetic diversity present in the wider COI dataset of FAW individuals available on GenBank (accessed Dec 2020). Six primers were employed in the FAW LAMP assay, two inner primers (FIP and BIP) and two outer primers (F3 and B3). The addition of loop primers (Floop and Bloop) facilitated a faster reaction. The optimised primer ratio (F3/B3: FIP/BIP: Floop/Bloop) was determined to be 1:6:3, with final primer concentrations of 0.4 µM, 2.4 µM and 1.2 µM, respectively.

**Table 1:** FAW COI LAMP primer and amplicon sequences (gBlock) and parameters.

LAMP primer or amplicon	Sequence 5′–3′	Primer Length (bp)	Predicted Tm, annealing temperature (°C)	Degeneracy of primer (fold)
FAW gBlock fragment	cccATGATACTTACTATGTAGTTGCTCATTTCgggCACTATGTTTTATCAATAGGAGCTGcccGCTATTTTAGGTGGATTTATTCACTGgggCCATTATTTACTGGATTATCTTTAAATCCgggCCTTATATATTAAAAATTCAATTTTTTATTATATTTATCcccGGAGTAAATTTAACTTTCTTCCCAgggTTTAGGATTAGCAGGTATACCTCGcccTGATTATCCTGATTCTTATATTTCATGAAccc	252	N/A	N/A
FAW_F3	ATGATACTTACTATGTAGTTGCTCATTTC	29	59.9	None
FAW_B3	TTCATGAAATATAAGAATCAGGATAATCA	29	62.4	None
FAW_FIP	GGATTTAAAGATAATCCAGTAAATAATGGCAYTATGTTTTATGAATAGGAGCTG	54	77.8	2
FAW_BIP	CCTTATWTATTAAAAATTCAATTTTTTATTATATTTATCCGAGGTATACCTGCTAAYCCTAAA	63	73.4	4
FAW_Floop	CARTGAATAAATCCHCCTAAAATAGC	26	60.9	4
FAW_Bloop	GGAGTAAATTTAACTTTYTTCCCA	24	60.2	2

The F2 and B2 primer regions of FIP and BIP are underlined. Lowercase letters in the gBlock indicate extra “ccc” or “ggg” added between LAMP primer sites to increase the overall Tm of the amplicon.

### FAW LAMP assay specificity results

**Figure 3:**
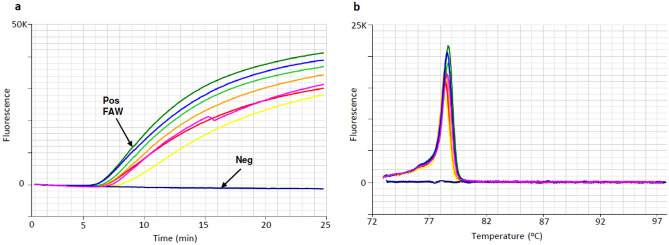
Optimised LAMP assay performed on FAW larva and adult moth laboratory DNA extracts. (**a**) Amplification profile, with 7 positive samples amplifying in approx. 10 min and negative sample (dark blue) showing a flat line. (**b**) Anneal derivative of LAMP amplicons, with an anneal derivative of 78.5 °C.

Positive LAMP reactions from FAW DNA amplified in less than 15 min, with anneal derivative temperatures of approx. 78.3 ± 0.3 °C (Table 3, Fig. 3). Amplification in less than 20 min was considered as positive. The specificity of the FAW LAMP assay was validated against a broad range of non-target taxa (Table 2, Fig. 1), with no off-target amplification observed within 20 min (Table 3). A small degree of off-target amplification was observed when reactions were run past 20 min (Table 3), hence the recommended cut-off time for positive amplification is 20 min. LAMP amplification was not sensitive to the DNA extraction method employed, with in-field compatible DNA extractions providing results consistent with laboratory DNA extractions (Table 4).

**Table 2:** Panel of Noctuidae (Lepidoptera) specimens tested for the FAW LAMP assay.

Species	Life stage	Source	Number	Common Name	Extraction method	GenBank Accession No	FAW LAMP*
***Spodoptera frugiperda***	**Adult moth**	**AgVic CHS**	**VAITC10705**	**Fall Armyworm**	**Destructive Qiagen**	**OL539296**	**+**
***Spodoptera frugiperda***	**Adult moth**	**AgVic CHS**	**VAITC10706**	**Fall Armyworm**	**Destructive Qiagen**	**OL539297**	**+**
***Spodoptera frugiperda***	**Larva**	**AgVic CHS**	**VAITC10707**	**Fall Armyworm**	**Destructive Qiagen**	**OL539298**	**+**
***Spodoptera frugiperda***	**Adult moth**	**AgVic CHS**	**VAITC10708**	**Fall Armyworm**	**Destructive Chelex**	**OL539299**	**+**
***Spodoptera frugiperda***	**Adult moth**	**AgVic CHS**	**VAITC10709**	**Fall Armyworm**	**Destructive Chelex**	**OL539300**	**+**
***Spodoptera frugiperda***	**Adult moth**	**AgVic CHS**	**VAITC10710**	**Fall Armyworm**	**Destructive Chelex**	**OL539301**	**+**
***Spodoptera frugiperda***	**Adult moth**	**AgVic CHS**	**VAITC10711**	**Fall Armyworm**	**Destructive Chelex**	**OL539302**	**+**
***Spodoptera frugiperda***	**Adult moth**	**QDAF**	**VAITC10713**	**Fall Armyworm**	**Destructive Qiagen**	**OL539303**	**+**
***Spodoptera frugiperda***	**Adult moth**	**QDAF**	**VAITC10714**	**Fall Armyworm**	**Destructive Qiagen**	**OL539304**	**+**
***Spodoptera frugiperda***	**Larva**	**QDAF**	**VAITC10724**	**Fall Armyworm**	**Destructive Qiagen**	**OL539314**	**+**
***Spodoptera frugiperda***	**Larva**	**QDAF**	**VAITC10725**	**Fall Armyworm**	**Destructive Qiagen**	**OL539315**	**+**
***Spodoptera frugiperda***	**Larva**	**QDAF**	**VAITC10726**	**Fall Armyworm**	**Destructive Qiagen**	**OL539316**	**+**
***Spodoptera frugiperda***	**Larva**	**QDAF**	**VAITC10727**	**Fall Armyworm**	**Destructive Qiagen**	**OL539317**	**+**
***Spodoptera frugiperda***	**Larva**	**QDAF**	**VAITC10728**	**Fall Armyworm**	**Destructive Qiagen**	**OL539318**	**+**
***Spodoptera frugiperda***	**Larva**	**QDAF**	**VAITC10729**	**Fall Armyworm**	**Destructive Qiagen**	**OL539319**	**+**
***Spodoptera frugiperda***	**Adult moth**	**AgVic CHS**	**VAITC10651**	**Fall Armyworm**	**Destructive Chelex**	**OL539292**	**+**
***Spodoptera frugiperda***	**Adult moth**	**AgVic CHS**	**VAITC10652**	**Fall Armyworm**	**Destructive Chelex**	**OL539293**	**+**
***Spodoptera frugiperda***	**Larva**	**QDAF**	**0–175600**	**Fall Armyworm**	**Destructive Bioline**	**OL539281**	**+**
***Spodoptera frugiperda***	**Larva**	**QDAF**	**0–175602**	**Fall Armyworm**	**Destructive Bioline**	**OL539282**	**+**
*Spodoptera cosmoides*	Larva	CSIRO		Black Armyworm	Destructive Qiagen	OL539270	−
*Spodoptera eridania*	Larva	CSIRO		Southern Armyworm	Destructive Qiagen	OL539271	−
*Spodoptera exigua*	Larva	AgVic CHS	VAITC10615	Beet Armyworm	Destructive Chelex	OL539288	−
*Spodoptera exigua*	Larva	CSIRO		Beet Armyworm	Destructive Qiagen	OL539272	−
*Spodoptera littoralis*	Larva	CSIRO		Cotton Leafworm	Destructive Qiagen	OL539273	−
*Spodoptera litura*	Adult moth	QDAF	VAITC10719	Taro Caterpillar	Destructive Qiagen	OL539309	−
*Spodoptera litura*	Adult moth	QDAF	VAITC10720	Taro Caterpillar	Destructive Qiagen	OL539310	−
*Spodoptera litura*	Larva	QDAF	VAITC10722	Taro Caterpillar	Destructive Qiagen	OL539312	−
*Spodoptera litura*	Larva	QDAF	VAITC10723	Taro Caterpillar	Destructive Qiagen	OL539313	−
*Spodoptera litura*	Larva	DAWE	VAITC10879	Taro Caterpillar	Destructive Chelex	OL539320	−
*Spodoptera litura*	Larva	QDAF	0–175619	Taro Caterpillar	Destructive Bioline	OL539283	−
*Spodoptera litura*	Larva	QDAF	0–175578	Taro Caterpillar	Destructive Bioline	OL539284	−
*Spodoptera mauritia*	Larva	QDAF	0–175608	Lawn Armyworm	Destructive Bioline	OL539285	−
*Spodoptera mauritia*	Larva	QDAF	0–175609	Lawn Armyworm	Destructive Bioline	OL539286	−
*Spodoptera ochrea*	Larva	CSIRO		N/A	Destructive Qiagen	OL539274	−
*Spodoptera ornithogalli*	Larva	CSIRO		Yellow Striped Armyworm	Destructive Qiagen	OL539275	−
*Dasygaster padockina*	Adult moth	AgVic CHS	VAITC9744	Tasmanian Cutworm	Non-destructive QE	OL539321	−
*Helicoverpa armigera armigera*	Larva	CSIRO	Haa-AD10	Cotton Bollworm	Destructive Qiagen	OL539263	−
*Helicoverpa armigera armigera*	Larva	CSIRO	Haa-AD13	Cotton Bollworm	Destructive Qiagen	OL539264	−
*Helicoverpa armigera conferta*	Larva	AgVic CHS	VAITC10314	Cotton Bollworm	Destructive Qiagen	OL539287	−
*Helicoverpa amigera conferta*	Adult moth	QDAF	VAITC10721	Cotton Bollworm	Destructive Qiagen	OL539311	−
*Helicoverpa armigera conferta*	Adult moth	AgVic CHS	VAITC10634	Cotton Bollworm	Destructive Chelex	OL539289	−
*Helicoverpa armigera conferta*	Larva	CSIRO	Hac	Cotton Bollworm	Destructive Qiagen	OL539266	−
*Helicoverpa assulta*	Larva	CSIRO	Hass2	Oriental Tobacco Budworm	Destructive Qiagen	OL539265	−
*Helicoverpa assulta*	Larva	CSIRO		Oriental Tobacco Budworm	Destructive Qiagen	OL539267	−
*Helicoverpa gelotopoeon*	Larva	CSIRO	Arg1	South American Bollworm	Destructive Qiagen	MG437199	−
*Helicoverpa gelotopoeon*	Larva	CSIRO	Arg2	South American Bollworm	Destructive Qiagen	OL539268	−
*Helicoverpa punctigera*	Adult moth	AgVic CHS	VAITC10650	Native Budworm	Destructive Chelex	OL539291	−
*Helicoverpa punctigera*	Larva	CSIRO		Native Budworm	Destructive Qiagen	OL539269	−
*Leucania loreyi*	Adult moth	QDAF	VAITC10715	False Armyworm	Destructive Qiagen	OL539305	−
*Leucania loreyi*	Adult moth	QDAF	VAITC10716	False Armyworm	Destructive Qiagen	OL539306	−
*Leucania loreyi*	Adult moth	QDAF	VAITC10717	False Armyworm	Destructive Qiagen	OL539307	−
*Leucania loreyi*	Adult moth	QDAF	VAITC10718	False Armyworm	Destructive Qiagen	OL539308	−
*Leucania loreyi*	Larva	QDAF	0–175593	False Armyworm	Destructive Bioline	OL539276	−
*Leucania loreyi*	Larva	QDAF	0–175594	False Armyworm	Destructive Bioline	OL539277	−
*Leucania stenographa*	Adult moth	AgVic CHS	VAITC10649	Sugarcane Armyworm	Destructive Chelex	OL539290	−
*Leucania stenographa*	Larva	QDAF	0–175611	Sugarcane Armyworm	Destructive Bioline	OL539278	−
*Mythimna convecta*	Larva	AgVic CHS	VAITC10679	Australian Armyworm	Destructive Chelex	OL539294	−
*Mythimna convecta*	Larva	AgVic CHS	VAITC10680	Australian Armyworm	Destructive Chelex	OL539295	−
*Mythimna convecta*	Adult moth	AgVic CHS	VAITC9749	Australian Armyworm	Non-destructive QE	OL539325	−
*Mythimna separata*	Larva	QDAF	0–175610	Paddy Armyworm	Destructive Bioline	OL539279	−
*Mythimna separata*	Larva	QDAF	0–175612	Paddy Armyworm	Destructive Bioline	OL539280	−
*Persectania dyscrita*	Adult moth	AgVic CHS	VAITC9746	Inland Armyworm	Non-destructive QE	OL539323	−
*Persectania dyscrita*	Adult moth	AgVic CHS	VAITC9747	Inland Armyworm	Non-destructive QE	OL539324	−
*Persectania ewingii*	Adult moth	AgVic CHS	VAITC9757	Southern Armyworm	Non-destructive QE	OL539326	−
*Persectania ewingii*	Adult moth	AgVic CHS	VAITC9758	Southern Armyworm	Non-destructive QE	OL539327	−
*Persectania ewingii*	Adult moth	AgVic CHS	VAITC9759	Southern Armyworm	Non-destructive QE	OL539328	−
*Persectania ervingii*	Adult moth	AgVic CHS	VAITC9760	Southern Armyworm	Non-destructive Q E	OL539329	−
*Proteuxoa cyanoloma*	Adult moth	AgVic CHS	VAITC9745	Two-spot Noctuid	Non-destructive QE	OL539322	−

Bold indicates the target species. “ + ” indicates FAW LAMP amplification within 20 min, “−“ indicates no amplification.

AgVic CHS, Agriculture Victoria Crop Health Services; CSIRO, Commonwealth Scientific and Industrial Research Organisation; DAWE, Department of Agriculture Water and Environment; QDAF, Department of Agriculture and Fisheries Queensland; QE, QuickExtract.

*See Results.

**Table 3:** Performance of the FAW LAMP assay using the optimised primer ratio 1:6:3.

Species	n	Amplification	Time (min)	Temperature (°C)
			Mean ± SD	Mean ± SD
*Spodoptera frugiperda*	19	19/19	9.2 ± 1.1	78.3 ± 0.3
*Spodoptera cosmoides*	1	0		
*Spodoptera eridania*	1	0		
*Spodoptera exigua*	2	0		
*Spodoptera littoralis*	1	0		
*Spodoptera litura*	7	1/7	24	None
*Spodoptera mauritia*	2	2/2	22	77.9 ± 0.2
*Spodoptera ochrea*	1	0		
*Spodoptera ornithogalli*	1	0		
*Dasygaster padockina*	1	0		
*Helicoverpa armigera armigera*	2	0		
*Helicoverpa armigera conferta*	4	2/4	24	77.6 ± 0.0
*Helicoverpa assulta*	2	0		
*Helicoverpa gelotopoeon*	2	0		
*Helicoverpa punctigera*	2	0		
*Leucania loreyi*	6	4/6	24 ± 0.1	77.8 ± 0.2
*Leucania stenographa*	2	1/2	22	None
*Mythimna convecta*	3	1/3	24	78.0
*Mythimna separata*	2	0		
*Persectania dyscrita*	2	0		
*Persectania ewingii*	4	0		
*Proteuxoa cyanoloma*	1	0

**Table 4:** In-field compatible non-destructive DNA extracted from a single thoracic leg of FAW caterpillar or moth using two different extraction buffers tested for FAW LAMP assay.

DNA extraction method	n	Time (min)	Temperature (^o^C)
		Mean ± SD	Mean ± SD
**QuickExtract**			
Larva	3	10.3 ± 0.7	78.6 ± 0
Adult	3	12.0 ± 1.3	78.7 ± 0
**Xtract**			
Larva	2	9.0 ± 1.1	78.5 ± 0.2
Adult	2	13.4 ± 0.5	78.6 ± 0

### Kim et al***.***^37^ LAMP assay results with published primers

The first set of primer master mix tested for this FAW LAMP assay on the Genie III using 1:6:3 ratio including all six primers (F3/B3, FIP/BIP and Floop/Bloop) and 65 °C amplification temperature, amplified all DNA samples tested, including the negative control, within one minute confirming a strong primer dimer (Supplementary Fig. 1a). The second set of primer master mix tested for this FAW LAMP assay on the Genie III using 1:8:2 ratio of five primers, as recommended by Kim, et al.^37^ (F3/B3, FIP/BIP and Bloop no Floop), and an amplification temperature of 61 °C resulted in no amplification after a 25 min reaction time. (Supplementary Fig. 1b).

### Detection sensitivity of gBlock DNA fragment

**Figure 4:**
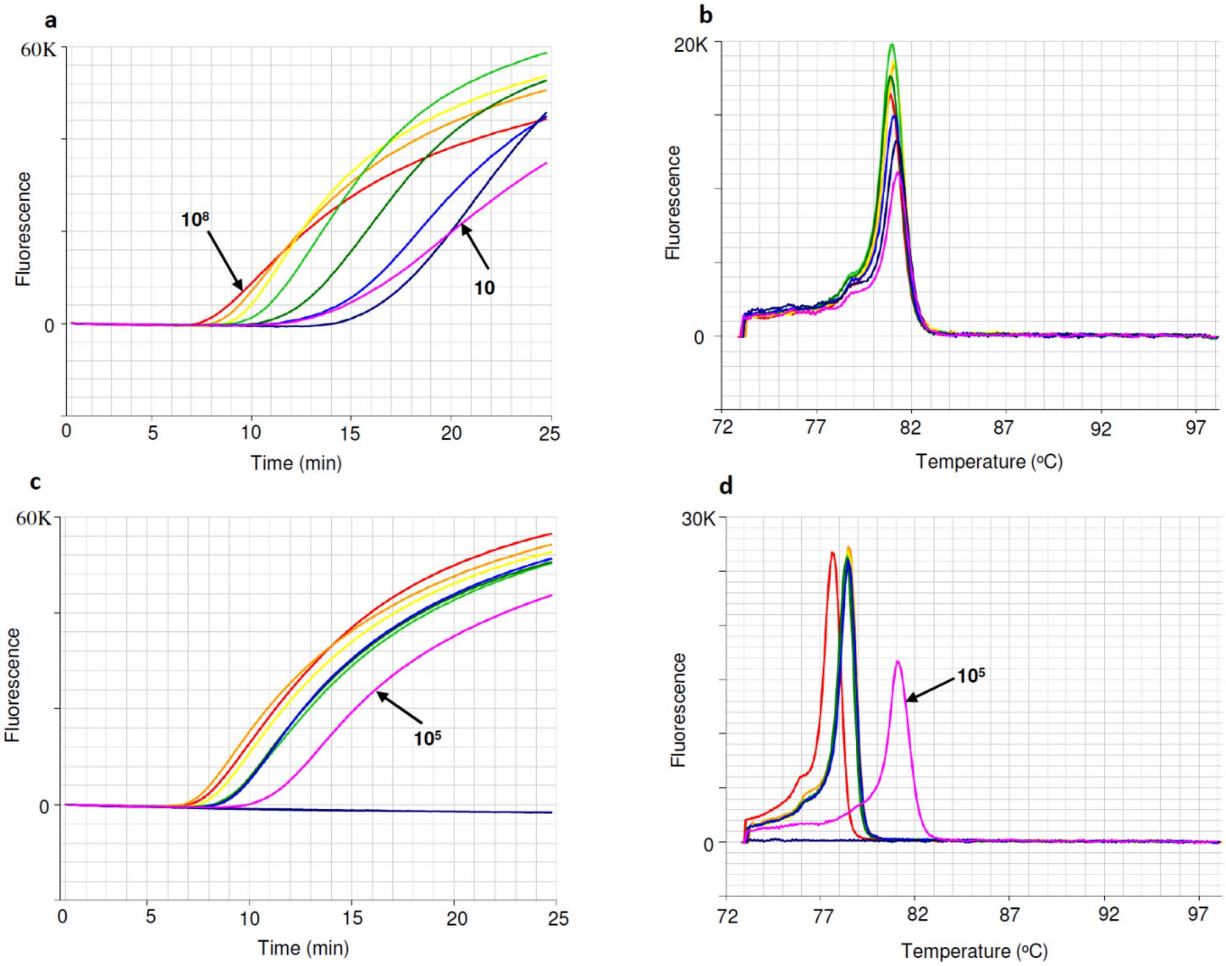
Detection sensitivity of FAW gBlock dsDNA amplicons (upper), evaluating amount of FAW DNA with gBlock DNA (lower). (**a**) Amplification profile with gBlock templates ranging from 10^8^ to 10 copies at ten-fold dilution. (**b**) Anneal derivative of gBlock LAMP amplicons, with an anneal derivative of 81 °C. (**c**) Amplification profile of four-fold dilution of FAW DNA (VAITC 10726) and gBlock DNA (10^5^ copies, pink). (**d**) Anneal derivative of LAMP amplicons showing two peaks, 78.5 °C for FAW DNA dilutions and 81 °C for gBlock DNA (pink).

The detection sensitivity of the FAW 252 bp gBlock dsDNA fragment (Table 1) was tested using ten-fold dilutions ranging from ~ 100 million copies to ~ 10 copies in LAMP reactions (Fig. 4), with positive detections found as low as ~ 10 copies within 20 min (Fig. 4a). The gBlock anneal derivative peak occurred at 81 °C (Fig. 4b). From the amplification profile it was calculated that one hundred thousand copies (10^5^) of gBlock DNA equates to less than 0.4 ng/µL of FAW DNA (Fig. 4c). The anneal derivative of LAMP amplicons exhibited two peaks, with the FAW DNA peak present at 78.5 °C and the gBlock DNA peak present at 81 °C (Fig. 4d). Alternatively, 10^6^ gBlock could be used as positive control which was found to amplify within 10–11 min.

### Colorimetric assay—new assay compared with Kim, et al.^37^ assay

**Figure 5:**
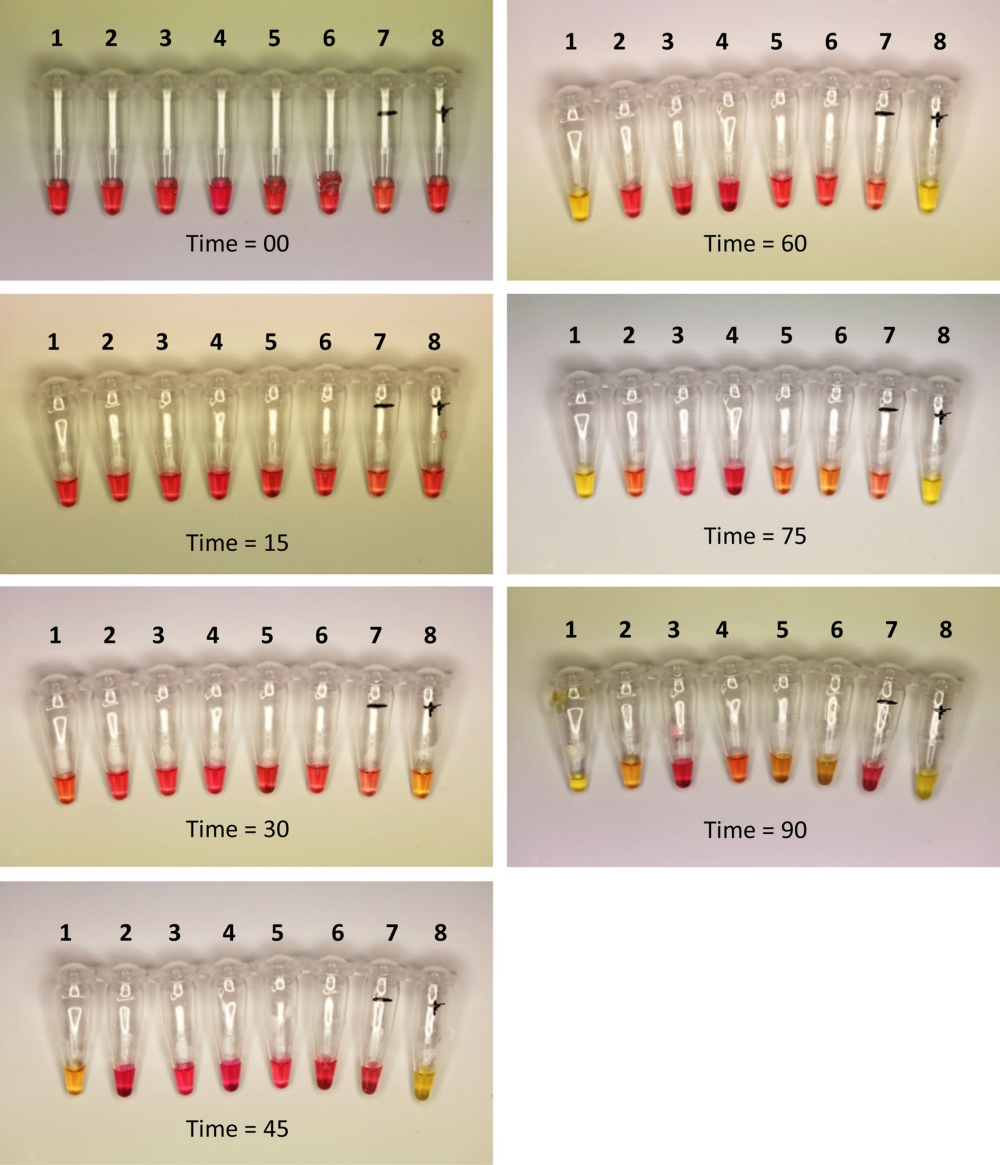
Time-series of FAW LAMP (new assay primers) using colorimetric master mix. Ninety minutes total amplification time shown in increments of 15 min. Samples: (1) *Spodoptera frugiperda*, (2) *Spodoptera litura* (PNG), (3) *Spodoptera exigua,* (4) *Helicoverpa armigera conferta,* (5) *Mythimna convecta,* (6) *Leucania loreyi,* (7) no-template negative control and (8) FAW gBlock DNA dilution 10^6^. The colour change from pink to yellow in tube 1 and 8 indicates positive samples. Negative samples did not change colour.

The LAMP colorimetric master mix can be used on a simple heating block (at 65 °C). Amplification using a colorimetric master mix (Fig. 5) was found to take significantly longer than the standard OptiGene reagents (which amplify within 20 min). Of six species tested in the colorimetric assay for the new FAW LAMP assay, only *S. frugiperda* and gBlock dilution 10^6^ produced a positive colour change in less than one hour, further demonstrating the robustness of our assay (Fig. 5). Optimal colorimetric results were achieved in 45 to 60 min for this assay. A small degree of non-target amplification was observed when the assay was run for longer (i.e., up to 90 min).

Following the Kim, et al.^37^ protocol, amplification using the colorimetric master mix (at 61 °C) was found to take approx. double the time to produce positive amplification (> 105 min). Of the six species tested in the colorimetric assay for the FAW LAMP assay, *S. frugiperda* produced a positive colour change in about 105 to 120 min (Supplementary Fig. 2). A small degree of non-target amplification was observed when the assay was run for longer (i.e., up to 165 min).

### Sensitivity of LAMP and real-time PCR assay

**Figure 6:**
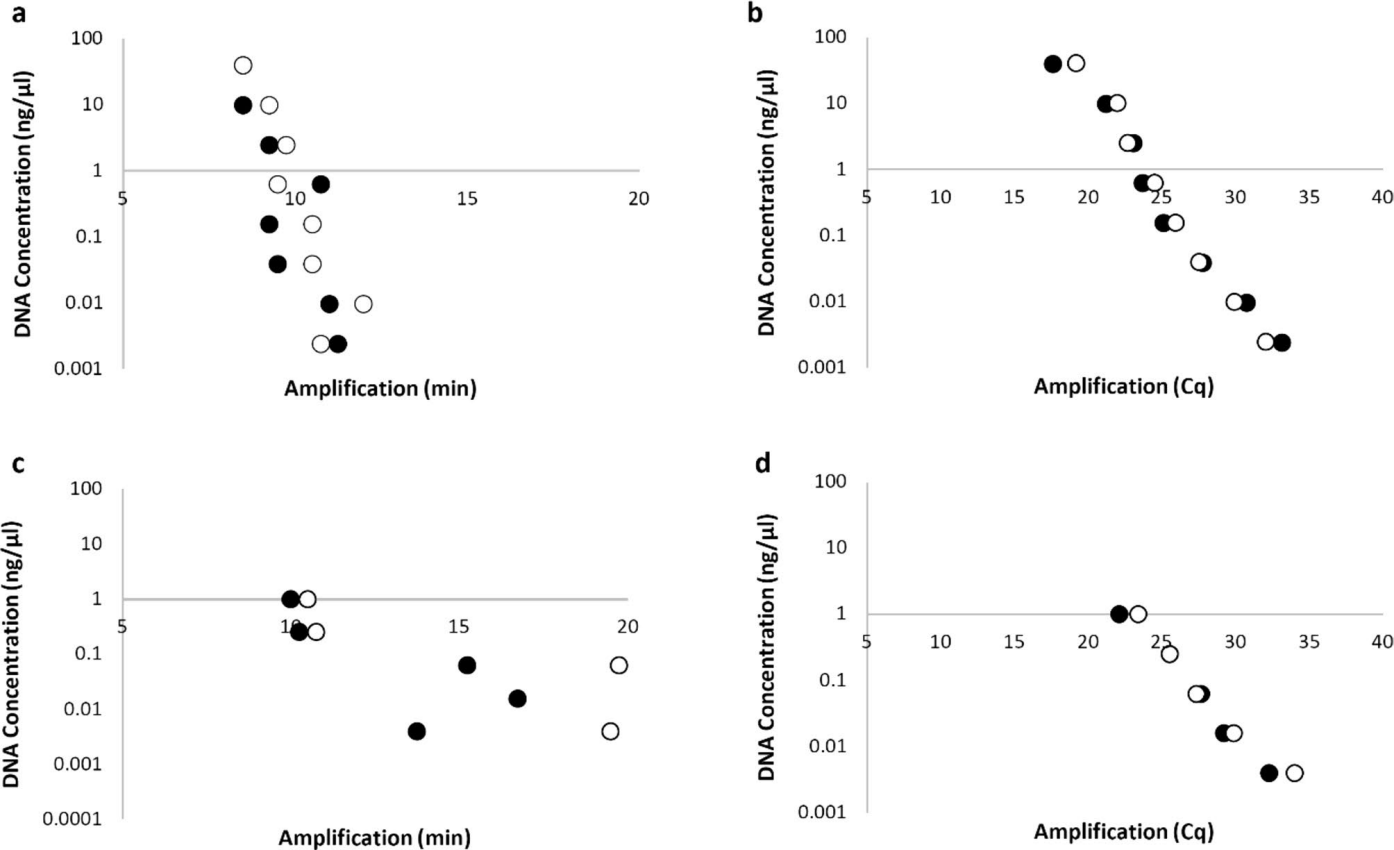
DNA sensitivity test of FAW LAMP and FAW real-time PCR assays. (**a,b**) A four-fold DNA dilution series of two biological replicates of FAW larvae (VAITC 10707 and 10726) DNA amount ranging from 40.0 ng/µL to 2.441 × 10^–3^ ng/µL. (**a**) FAW LAMP assay amplification time, sensitive to all 8 DNA dilutions tested. (**b**) Real-time PCR Cq values sensitive to all 8 dilutions tested. (**c,d**) A four-fold DNA dilution series of two biological replicates of FAW adult moth (VAITC 10728 and 10729) DNA amount ranging from 1.0 ng/µL to 6.1 × 10^–5^ ng/µL. (**c**) FAW LAMP assay amplification time, sensitive to only 5 out of 8 DNA dilutions tested. (**d**) Real-time PCR Cq values sensitive to only 5 out of 8 DNA dilutions tested. Black and white circles represent biological replicate DNA samples.

The sensitivity of the new FAW LAMP assay (Fig. 6a) and real-time PCR assay (Fig. 6b) were tested using a four-fold serial dilution of DNA for biological replicates of late-instar larval thoracic leg. A four-fold serial dilution of DNA for biological replicates of an adult moth leg was tested with the new FAW LAMP assay (Fig. 6c) and real-time PCR assay (Fig. 6d). The results from both assays were very similar, both proving to be very sensitive. Both LAMP and real-time PCR assays was able to detect FAW DNA from larvae down to the lowest dilution tested 2.4E−03 ng/µL (equal to 2.4 pg) (Fig. 6a,b). Both LAMP and real-time PCR assays was able to detect FAW DNA from adult moths down to 5 out of 8 dilutions only 3.9E−03 ng/µL (equal to 3.9 pg) (Fig. 6c,d). Both LAMP and real-time PCR results produced similar results as the starting DNA amount for a late-instar larval thoracic leg was found to be approx. 40 times higher than from an adult moth leg.

## Discussion

This study reports on a LAMP assay for rapid and reliable in-field detection of fall armyworm (FAW, *Spodoptera frugiperda*), an invasive noctuid pest at both adult and larval stages. Our assay has been shown to be species-specific, when tested against a panel of twenty commonly encountered noctuids, including approx. a third of all known *Spodoptera* species (i.e. 9 of the 31 species^39^). Our primers were capable of amplifying positive FAW DNA in under 10 min, with amplification within 20 min considered positive. We recommend that extended LAMP amplification not be performed, as a small degree of off-target amplification was observed when reactions were run past 20 min. We also designed and optimised a synthetic DNA positive control (gBlock dsDNA fragment) for use in our FAW LAMP assay. This gBlock is beneficial in providing a consistent control to allow tracking of the performance of LAMP assays across runs and provides confidence that positive amplification of samples is not due to contamination, as the gBlock DNA has a different anneal derivative temperature compared to FAW DNA. The new FAW LAMP assay described here has also been shown to perform well using the technologically simpler colorimetric approach, with amplification of positive FAW DNA occurring in less than an hour.

In its native New World geographic range, the FAW is widely considered to consist of either the corn or the rice host-preferred strains^20^. At the whole genome level however and based on the widely applied partial mitochondrial COI nucleotide distance estimates, these rice and corn-host strains could potentially be regarded as two closely related sister species^40,41^. However, whole genome analyses of invasive populations in the Old World showed FAW to consist of admixed genome signature overall, indicating that these were generally hybridised populations^42–44^. Our LAMP assay was developed to accurately identify all FAW, incorporating the range of COI DNA sequence variation known from *S. frugiperda, *sensu lato, regardless of their rice / corn host preferences, or if they represented hybridised individuals*.*

While the FAW LAMP assay developed here showed rapid and robust confirmation of FAW regardless of host strains or hybrids, the DNA data generated also unexpectedly revealed incongruency in the taxonomic status of some non-target Noctuidae species. For example, our DNA sequencing of *S. exigua* revealed that the two specimens collected in Africa and Australia are genetically divergent (4.4%, Fig. 1) and likely represent two discrete cryptic species, despite both matching with 100% similarity to different reference sequences identified as *S. exigua* in the Barcode of Life DNA (BOLD) and GenBank databases. Likewise, the generic designation of *Leucania loreyi*, which is commonly erroneously placed within the genus *Mythimna* in many studies, is also unclear^45^. When the geographic distribution of both *L. loreyi* and *M. loreyi* are combined this species is very widespread (GBIF, accessed 20-Sept 2021), however confirmation of the taxonomic status for this widespread Old World pest is required to afford confidence in the LAMP assay which has recently been developed for *M. loreyi*^46^.

For both pre-border interception and in-field applications, all FAW life stages are expected to be encountered, including egg-masses and early-instar larvae. Laboratory testing of the new LAMP assay on such samples was not possible here, as they were not available. However, other molecular approaches, such as PCR–RFLP which has been used to differentiate *Helicoverpa* species^47^, have been shown to be effective on these early life stages. Additionally, LAMP assays for other insects have been shown to work on all lifestages^30,31^ and given the sensitivity of our FAW LAMP assay (i.e., down to ~ 10 copies of gBlock DNA fragments and 2.4 pg of FAW DNA) it is anticipated that the assay outlined here will also be able to accurately identify these early life stages of FAW.

The comparison of the published Kim, et al.^37^ LAMP assay revealed that our new assay is the most suitable for in-field use, being capable of producing results more rapidly in a portable real-time fluorometer, using appropriate commercially available reagents, or using the alternative colorimetric approach. As LAMP amplification times are influenced by both amplicon length and the presence of loop primers^48^, it is likely that our new assay produces such rapid amplification times (approx. 10 min) due to it consisting of a relatively small amplicon fragment and employing two loop primers. The second published FAW LAMP assay^38^ also does not use loop primers, but was not directly compared in our study. This latter assay has also been shown to be capable of rapidly amplifying FAW DNA using the GenieIII, and has been tested against larvae of eleven non-target Noctuidae, including two *Spodoptera* species to-date^38^.

Prevention of on-going introduction of novel economically significant traits in new invasive pest populations is a biosecurity priority^2,49^. FAW has been shown to have diverse insecticide and Bt resistances^10,50,51^. Currently, there are no rapid in-field molecular tools available to screen for resistance in FAW, although these may be developed in the future. For now, our LAMP assay provides a new tool to aid in monitoring FAW incursions, through providing rapid, accurate species identification, using simple protocols for DNA extraction and LAMP amplification including a gBlock positive control, being capable of being performed on a portable real-time fluorometer in the field.

## Materials and methods

### Specimens examined

Adult and larval specimens of the FAW target species, *Spodoptera frugiperda* (n = 19 individuals), and non-target Noctuidae species (n = 20 species, n = 49 individuals), were examined in this study (Table 2). All species identities were confirmed through DNA barcoding of the mitochondrial COI locus (5′-region) following standard laboratory procedures^52,53^.

### DNA extractions

DNA was extracted from single (thoracic) legs removed from adult and late-instar larval Noctuidae specimens (Table 2), in the laboratory using the DNeasy Blood and Tissue extraction kit (Qiagen, USA); the ISOLATE II Genomic DNA Kit (Bioline, UK); and the 5% Chelex 100 (BioRad, USA) extraction method, all following manufacturer recommendations and standard laboratory protocols^54^. DNA was quantified either by a NanoDrop ND-1000 Spectrophotometer (Thermo Fisher, Australia) or a Qubit 2.0 Flourometer (Invitrogen, Life Technologies, Australia) and subsequently stored at − 20 °C.

In-field compatible extraction procedures employed were either the QuickExtract™ (QE) solution 1.0 (Epicentre Biotechnologies, USA) (as per Blacket, et al.^30^), or the Xtract (Xt) DNA extraction solution (GeneWorks, Australia) as follows: 50 µL of extraction solution was pipetted into each well of an 8-well Genie strip (OptiGene, UK) with one leg of FAW adult moth (n = 3) and one leg of FAW larva (n = 3) and incubated in the Genie III at 65 °C for 6 min, followed by 2 min at 98 °C^30^, and then kept on ice for > 1 min.

### Development of a *Spodoptera frugiperda* (FAW) LAMP assay

#### LAMP primer design

Six novel LAMP primers were manually designed by eye to target eight DNA regions from a COI reference alignment (3′-COI region), including twenty-three *Spodoptera* species (from Kergoat, et al.^55^ Nagoshi, et al.^20^). The reference alignment was compiled from existing DNA sequences of *Spodoptera* species available on GenBank (accessed Dec 2020), to the mitochondrial genome from Kim, et al.^37^. The 3′-COI region was used for primer design as it was found that there were a larger number of DNA sequences available for this region than the standard 5′-COI DNA barcoding region for FAW and related Spodoptera species. The 3′-COI region has also been previously used for identification of host-specific strains within FAW^20^.

For all primers, the GC content (%), predicted melting temperature (Tm), and potential secondary structure formations (hairpins or dimers) were analysed using the Integrated DNA Technologies (IDT) online OligoAnalyzer tool (https://sg.idtdna.com/calc/analyzer), using the qPCR parameter sets. Complete sets of LAMP primers were analysed together to detect potential primer dimer interactions using the Thermo Fisher Multiple Primer Analyzer tool (www.thermofisher.com). Primers were synthesised by Sigma (Australia).

#### LAMP assay optimisation

Optimisation was performed following the protocols previously outlined in Blacket, et al.^30^, which include testing multiple primer ratios to obtain optimum amplification time and a consistent anneal derivative temperature. Primers F3 and B3 were used at 10 µM concentration, whilst FIP, BIP, Bloop and Floop were used at 100 µM concentration. The primer master mix was prepared to a ratio 1:6:3 by adding 10 µL of F3 and B3; 6 µL of FIP and BIP; 3 µL of Bloop and Floop; and 62 µL of ultrapure water, for a total volume of 100 µL. Each LAMP reaction mix was made by adding 10 µL of primer master mix to 14 µL of Isothermal Master Mix (ISO-004, OptiGene, UK) and 1 µL of template DNA into each well of the Genie strip (25 µL total reaction volume). Each run included a positive control (i.e., known FAW DNA, VAITC 10707), a no-template negative control, and six test samples.

LAMP assays were run in the Genie III at 65 °C for 25 min followed by an annealing curve analysis from 98 to 73 °C with ramping at 0.05 °C/s, visualised in the blue channel. LAMP runs taking approx. 35 min in total. The run date, Genie III serial number and the run number of each LAMP assay completed on the machine were recorded to allow run files to be transferred and analysed using a PC version of the software Genie Explorer version 2.0.7.11. The amplification and anneal derivative curves were visualised on the Genie III screen to ensure that amplification occurred as expected, with positive amplification plots showing an ‘S’ shaped sigmoid curve reflecting the increase in fluorescence detected, and negative results staying relatively flat. Positive results were further confirmed through performing the annealing step which results in a single product peak at a specific temperature.

### Validation of Kim, et al.^37^ primers on Genie III

The first set of primers tested were prepared in a master mix that included all six primers F3/B3, FIP/BIP and Floop/Bloop to a ratio of 1:6:3. The reaction was run on the Genie III using the OptiGene mastermix (ISO-004, OptiGene, UK) at 65 °C for an extended time of 35 min. The second primer set (colorimetric assay primers) tested consisted of only five primers F3/B3, FIP/BIP and Bloop (i.e. no Floop) at a ratio of 1:8:2 (as per Kim, et al.^37^). The reaction was run on the Genie III using the OptiGene mastermix at 61 °C for 25 min.

Both 5 and 6-primer sets were tested in two LAMP runs with OptiGene reagents as mentioned above using DNA templates in well 1 to 8 as listed sequentially. The first well containing DNA of the target species (1) *S. frugiperda*, (2) *Spodoptera litura* (Papua New Guinea, PNG), (3) *Spodoptera exigua*, (4) *Helicoverpa armigera conferta,* (5) *Mythimna convecta*, (6) *Leucania loreyi,* (7) no-template negative control and (8) FAW gBlock dilution 10^6^.

### Evaluation and design of a gBlock dsDNA fragment for the FAW LAMP assay

We designed a gBlock dsDNA fragment (Integrated DNA Technologies, Iowa, USA) for use as a positive control for the FAW LAMP assay. This DNA fragment consisted solely of concatenated LAMP primers separated by runs of “ccc” and “ggg” to increase the overall Tm of the fragment, and therefore, a different annealing derivative when compared to positive FAW samples.

Sensitivity of the LAMP assay was tested using the serially diluted gBlock DNA. The copy number calculation and ten-fold serial dilution (1:10) of the gBlock was prepared as outlined in^31^. Serial dilutions ranged from ~ 100 million copies down to ~ 10 copies (10^8^ copies to 10 copies) and were run in the Genie III following the same FAW LAMP protocol as previously mentioned. Following this, a second FAW LAMP run was conducted to determine the best dilution to be used as a positive control. The four-fold serial dilution of laboratory extracted FAW larva DNA (VAITC 10726) (40 ng/µL to 0.0391 ng/µL) was used as DNA template to compare with one hundred thousand copies (10^5^) of gBlock DNA. The amount of FAW DNA was then equated from the amplification time of FAW gBlock dilution 10^5^.

### Colorimetric FAW LAMP assay with new and published primers

We also tested our FAW LAMP assay primers using an alternative colorimetric LAMP master mix (WarmStart Colorimetric LAMP 2 × master mix, DNA & RNA, New England Biolabs Inc.) following published protocols^32^.

The volume of primer master mix in each reaction mixture was initially optimised using 2.5, 5 and 10 µL of primer mix. Ten microlitres of primer mix added to the reaction was able to produce colour change from pink to yellow in the shortest time. The reactions were set up in a 25 µL reaction volume and included 12.5 µL of colorimetric master mix, 10 µL of primer mix (1:6:3, F3/B3: FIP/BIP: Floop/Bloop which is the same primer mix as used for new FAW LAMP assay), 1 µL of template DNA (same order as mentioned in validation of Kim, et al.^37^ primers) and 1.5 µL of water, respectively.

Simultaneously, we tested the published colorimetric LAMP assay by Kim, et al.^37^ using their LAMP primers and published protocol. The reaction was set up in a total volume of 25 µL. Each reaction mixture included 12.5 µL of colorimetric master mix 2.5 µL of primer mix (1:8:2, F3/B3: FIP/BIP: Bloop only, no Floop), 1 µL of template DNA (same order as mentioned in validation of Kim et al*.* (2021) primers) and 9 µL of water, respectively.

We ran both assays side-by-side for comparison, showing a clear timeline for each test. The tubes were incubated on a heat block at 65 °C (our assay) and 61 °C (Kim, et al.^37^ assay) and the colour change was monitored by photographing with a Canon 5D digital SLR camera every 15 min for 90 min (our assay) and 165 min (Kim, et al.^37^ assay).

### Analytical sensitivity of the FAW LAMP assay compared to real-time PCR^28^

A four-fold serial dilution (1:4) of biological replicates of FAW late-instar larval thoracic leg (reference specimens VAITC 10707 and 10726) and FAW adult moth leg (reference specimens VAITC 10728 and 10729) laboratory extracted DNA was prepared using Ultrapure water (Invitrogen, Life Technologies, Australia). Starting DNA concentration was quantified using a Qubit 2.0 Flourometer (Invitrogen, Life Technologies, Australia) following manufacturers protocol. The DNA sample was serially diluted from 40.0 to 2.441 × 10^–3^ ng/µL (1:1 to 1:16,384) for the FAW larval DNA and 1.0 ng/µL to 6.1 × 10^–5^ ng/µL (1:1 to 1:16,384) for the FAW adult DNA. Sensitivity of the LAMP assay was tested for each of the four samples using the eight serially diluted DNA samples in the Genie III, following the same assay conditions as described above. The time of amplification and anneal derivative temperature was recorded for all samples.

The same serial dilution of DNA from above was compared for FAW DNA sensitivity using a real-time PCR assay. The primers and probe set (Sigma) and cycling conditions used were as published in^28^ except that the primer concentration was increased from 0.3 µM to 0.5 µM and probe concentration from 0.1 to 0.2 µM for optimum amplification. Real-time PCR was performed in QuantStudio 3 Real time PCR system (Thermo Fisher Scientific) in a total volume of 25 µL with technical replicates for each dilution. Each reaction mixture included 12.5 µL GoTaq Probe qPCR mastermix (Promega), 0.5 µM of each forward and reverse primers, 0.2 µM Taqman probe, 4 µL of template DNA and made up to 25 µL with RNA-free water. A non-template control with 4 µL of water instead of DNA was included in each run to check for reagent contamination. The PCR thermal cycling conditions consisted of a one-step denaturation: 2 min at 95 °C, followed by 40 cycles of amplification in a two-step procedure: 95 °C for 15 s and 60 °C for 1 min. The average Cq value (cycling quantification value) of the eight dilutions was recorded for comparison with the amplification time from the LAMP assay.

## Supplementary Information

Supplementary Information.

## Data Availability

GenBank, accession numbers OL539263 - OL539329.
