# Supplementary material for: A diagnostic LAMP assay for rapid identification of an invasive plant pest, fall armyworm Spodoptera frugiperda (Lepidoptera: Noctuidae)
Source: Sci Rep. 2022 Jan 21;12:1116. doi: 10.1038/s41598-021-04496-x (PMC8782856; doi:10.1038/s41598-021-04496-x)
Supplement: Supplementary file 1 — Supplementary Information. [file 41598_2021_4496_MOESM1_ESM.pdf]

A diagnostic LAMP assay for rapid identification of an invasive plant pest, fall armyworm *Spodoptera frugiperda* (Lepidoptera: Noctuidae)

Arati Agarwal, Lea Rako, Mark K. Schutze, Melissa L. Starkie, Wee Tek Tay, Brendan C. Rodoni & Mark J. Blacket

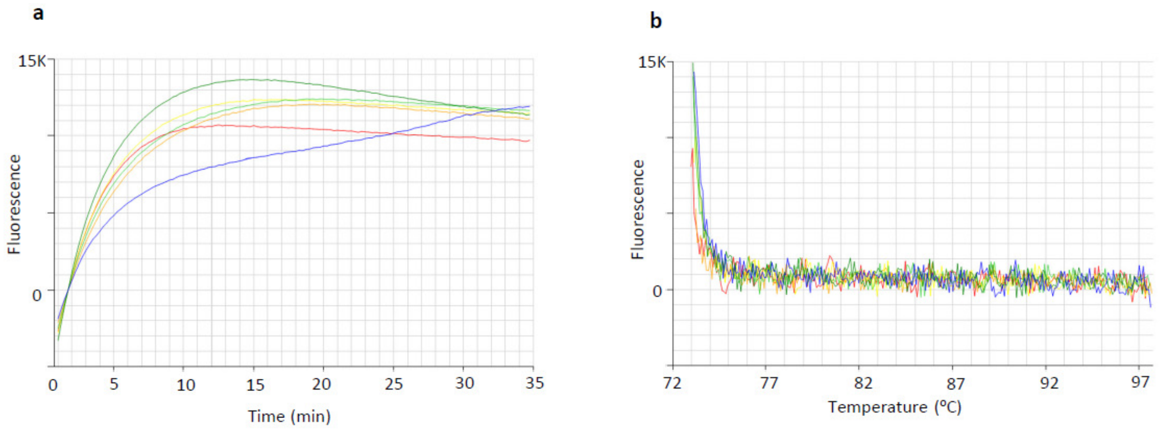

Figure S.1a. LAMP assay performed on Genie III using Kim *et al.* protocol with six primer ratio 1:6:3 and amplification at 65 °C. **a)** Amplification profile, all samples amplified including negative control (blue) indicating strong primer dimer **b)** Anneal derivative curve of LAMP amplicons – no specific anneal derivative temperature.

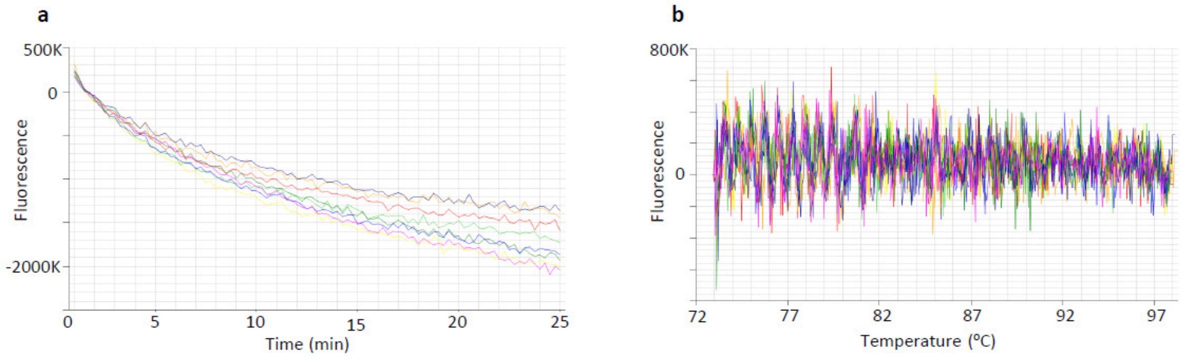

Figure S.1b. LAMP assay performed using Kim *et al.* protocol with five primers (no Floop) colorimetric primer ratio 1:8:2 and amplification at 61 °C. **a)** Amplification profile, none of the samples amplified due to absence of Floop primer. **b)** No anneal derivative curve of LAMP amplicons.

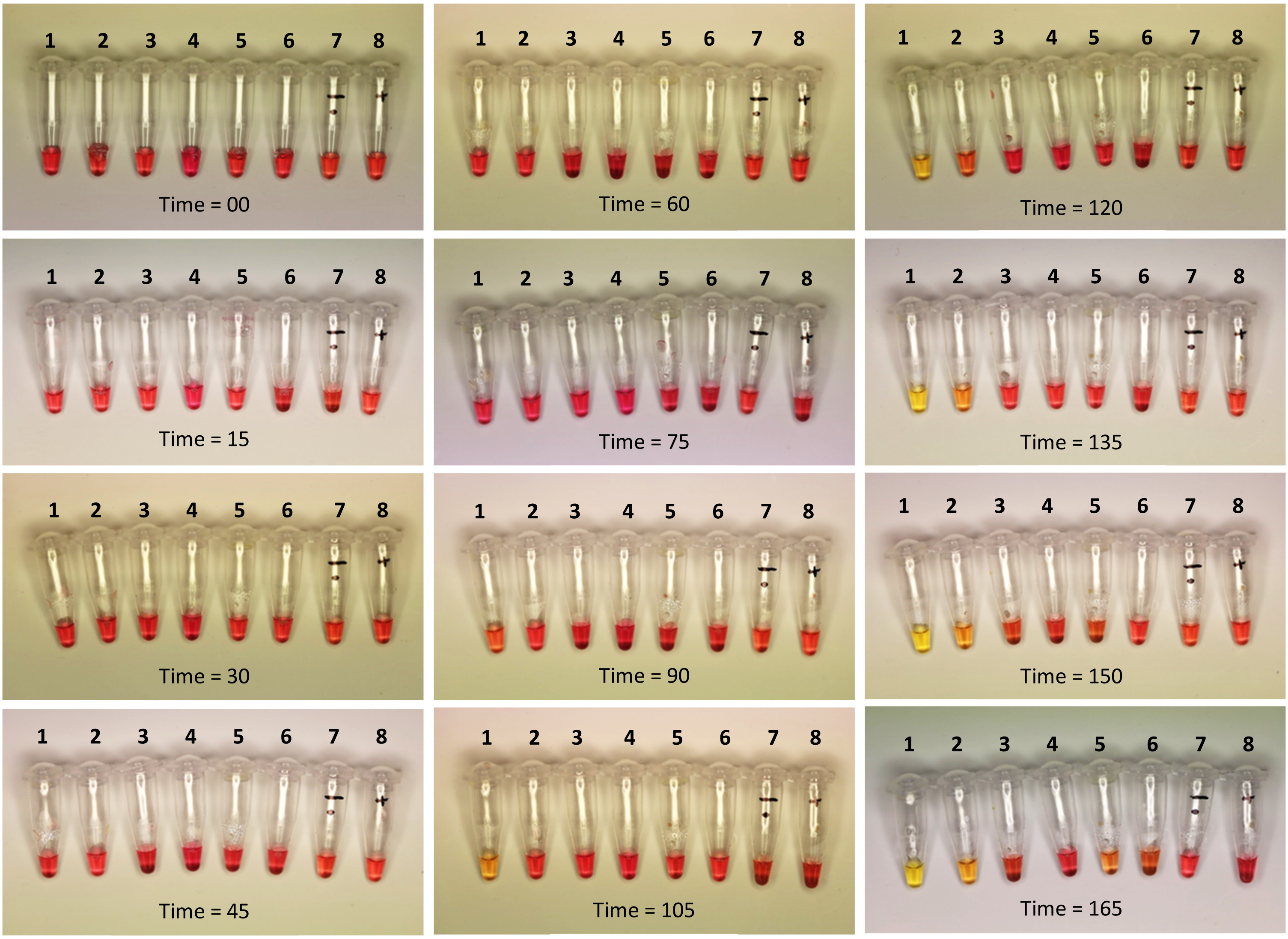

Figure S.2. Time-series of FAW LAMP (Kim *et al.*) using colorimetric master mix. One hundred- and sixty-five-min total amplification time shown in increments of 15 minutes. Samples: 1) *Spodoptera frugiperda*, 2) *Spodoptera litura (PNG),* 3) *Spodoptera exigua,* 4) *Helicoverpa armigera conferta,* 5) *Mythimna convecta,* 6) *Leucania loreyi,* 7) no-template negative control and 8) FAW gBlock dilution 10^6. The colour change from pink to yellow in tube 1 indicates positive sample. Negative samples did not change colour.

Reference:

37 Kim, J. et al. Development of a simple and accurate molecular tool for *Spodoptera frugiperda* species identification using LAMP. Pest Management Science **77,** 3145-3153 (2021).
